# The Incidence of Norovirus-Associated Gastroenteritis Outbreaks in Victoria, Australia (2002–2007) and Their Relationship with Rainfall

**DOI:** 10.3390/ijerph7072822

**Published:** 2010-07-05

**Authors:** Leesa D. Bruggink, John A. Marshall

**Affiliations:** Victorian Infectious Diseases Reference Laboratory, 10 Wreckyn Street, North Melbourne, Victoria 3051, Australia; E-Mail: leesa.bruggink@mh.org.au

**Keywords:** norovirus, rainfall, lag, outbreaks

## Abstract

The relationship between the incidence of norovirus-associated gastroenteritis outbreaks (NAGOs) in Victoria, Australia for the period 2002–2007 and rainfall was examined. Statistical analysis involving the correlation between time series indicated that there was a statistically significant (p < 0.05) correlation between monthly NAGO incidence and average monthly rainfall. There was a lag of an average of about three months between peak average rainfall and a NAGO epidemic. The findings thus indicate rainfall can influence NAGO incidence. In an era where there is concern about the potential effects of global warming on weather patterns, it should be borne in mind that future changes in NAGO incidence may reflect altered world weather patterns.

## 1. Introduction

Human infectious diseases sometimes show a seasonal periodicity [1,2]. One possible explanation is that there is a relationship between variables such as temperature, rainfall, and humidity and the incidence of a particular infectious disease. Studies in this area include, for example, work on arbovirus infections [3], general respiratory infections [4] and rotavirus [5,6].

The noroviruses, which are single-stranded positive-sense RNA viruses classified as the genus *Norovirus* within the family *Caliciviridae***,** are now considered the most common cause of outbreaks of non-bacterial gastroenteritis [7]. Studies on norovirus-associated gastroenteritis outbreaks (NAGOs) in the state of Victoria, Australia, indicate they tend to peak in warmer months of the year [8,9].

NAGO periodicity in the state of Victoria, Australia, appears to be related to two sets of controlling factors [9]. There is evidence that one of these is genetic [10] and it has been postulated that the other is environmental [9]. Currently, there is little understanding of what environmental factors may correlate with NAGO epidemics but there is evidence that temperature is not one of these factors, as NAGO epidemics tend to occur in colder months of the year in the northern hemisphere [11] and warmer months of the year in the southern hemisphere [8,9]. On the other hand, there is evidence that noroviruses can be spread by waterborne methods [12,13], thereby suggesting rainfall may be able to play a role in the transmission of NAGOs. This study therefore examined the relationship between the incidence of NAGOs and rainfall in Victoria, Australia for the period 2002–2007.

## 2. Materials and methods

### 2.1. Outbreak Definition and Investigation

For the purposes of this study an outbreak of gastroenteritis was defined as an incident, apparently associated with a common event or location, in which four or more individuals had symptoms of gastroenteritis.

The outbreaks included in this study were those for which specimens were sent to the Victorian Infectious Diseases Reference Laboratory (VIDRL) for testing in the period 2002–2007. VIDRL is the main public health laboratory for viral identification in the state of Victoria, Australia and, as such, it receives faecal material from gastroenteritis outbreaks reported to the Victorian Department of Human Services (DHS) where a viral aetiology is suspected. Outbreak specimens are also occasionally sent by other institutions such as hospitals. Only outbreaks which occurred in the state of Victoria, which is in the south-eastern corner of continental Australia, were included in the study.

The date of the outbreak was taken as the onset date if provided. If this was unavailable, the date taken was the date the outbreak was first notified to DHS or the earliest date of collection of a specimen for testing. Outbreak documentation and management were carried out by staff of DHS and/or staff at the affected setting. Only one faecal specimen per person per outbreak was tested for norovirus.

### 2.2. Laboratory Studies

Faecal specimens were prepared as a 20% (vol/vol) suspension in Hanks’ complete balanced salt solution, and the suspension vigorously shaken and then clarified by centrifugation [14]. The clarified fluid was then collected for RNA extraction. RNA extraction was carried out using either the manual Qiagen QIAamp viral RNA mini-kit cat. No. 52906 (Qiagen GmbH, Hilden, Germany) [15] or the Corbett (Corbett Robotics Pty. Ltd., Eight Mile Plains, Qld., Australia) automated nucleic acid extraction procedure [15]. Open reading frame 1 reverse transcription-polymerase chain reaction testing for norovirus was carried out essentially as given by Yuen *et al.* [16].

### 2.3. Data Analysis

Weather information was derived from the Australian Bureau of Meteorology (ABM), either from their website (home page: http://www.bom.gov.au/) or from information provided for this study by the ABM. The rainfall data used were the average monthly rainfall (mm) for the whole state of Victoria.

### 2.4. Statistical Analysis

The relationship between monthly NAGO incidence and monthly rainfall was investigated by the method of time series correlation [17]. Before correlation between pairs of time series was investigated, three preliminary procedures were necessary and were systematically applied to each of the time series being considered. The preliminary procedures were necessary to obtain a fit for each time series such that the autocorrelation coefficients ([18], pp. 18–20) of the residuals of the fit ([19], p. 530) were not statistically significant. Firstly, as the year to year variation was not relevant to the study, it was eliminated by using a suitable filter ([18], pp. 13–15). Secondly, the month to month variation was accounted for by fitting an equation by least squares using dummy variables as described by Levine *et al.* ([19], pp. 700–706). In this way the annual cycle was removed from the data. Thirdly, the first 18 autocorrelation coefficients of the residuals from the fit were calculated as described by Chatfield ([18], pp. 49–50) and the autocorrelation coefficients were inspected to see if any were significant at the p = 0.05 level. The test used was the more accurate of the two tests suggested by Chatfield ([18], p. 51). When any were significant, autoregression terms ([19], pp. 684–693) were added to the fit to ensure that all the autocorrelation coefficients of the residuals were not significant ([18], p. 62).

The method of Haugh [17] was then applied to the residuals so calculated of the two time series being compared. The cross-correlation coefficients were calculated by the method of Chatfield ([18], pp. 138–139). If any of the cross-correlation coefficients were significant (p < 0.05) following the criterion in Section 5 of Haugh [17], then the two time series were significantly correlated with each other and were not independent of each other.

## 3. Results

### 3.1. General

During the period 2002–2007, 8,507 faecal specimens from 1,495 gastroenteritis outbreaks were tested. One or more specimens per outbreak were positive for norovirus in 1,018 outbreaks (*i.e.*, 1,018 NAGOs were identified).

**Figure 1:**
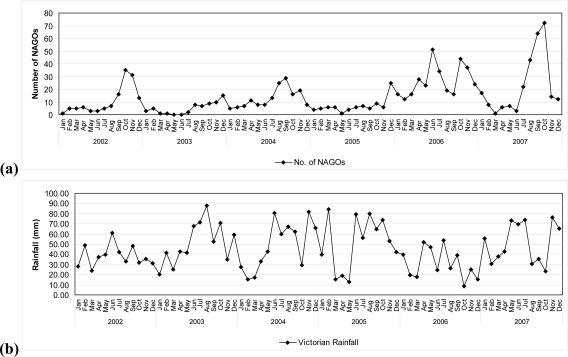
(a) Incidence of NAGOs per month in Victoria, 2002–2007. (b) Average monthly rainfall for Victoria, 2002–2007.

The periodicity of NAGOs during the period 2002–2007 is given in Figure 1 (a). It can be seen that for each calendar year there was a marked peak towards the end of each year except that that in 2006 there was an additional peak in the middle of the year. Each such peak will be referred to as a “NAGO epidemic”. For purposes of analysis, a NAGO epidemic was defined as occurring in the three consecutive months of highest NAGO incidence.

### 3.2. NAGO Incidence and Rainfall

The relationship between NAGOs and rainfall is given in Figure 1. Application of the statistical procedure of Haugh [17] confirmed there was a relationship between NAGO incidence and rainfall. A significant (p < 0.05) cross-correlation coefficient was found so that the two time series (*i.e.*, NAGOs and rainfall) were significantly related.

### 3.3. The Time Lag between Highest Rainfall and NAGO Epidemics

The time lag between the three-month period of highest rainfall and the NAGO epidemic(s) in each calendar year is given in Table 1. It was found that there was a time lag of an average of about three months between the midpoint of the three-month period of highest rainfall and the midpoint of the NAGO epidemic(s) following in the same calendar year.

## 4. Discussion

Norovirus infections tend to occur more commonly in cooler months of the year in some northern hemisphere countries [11], but tend to occur more commonly in warmer months in a southern hemisphere region such as the state of Victoria, Australia [8,9]. However, the relationship between NAGOs and weather has not been systematically investigated and the current study is the first detailed attempt to establish a link between NAGOs and rainfall.

The results of the current study indicate NAGO incidence can be influenced by rainfall. In the first place, a statistically significant correlation was found between monthly NAGO incidence and average monthly rainfall. This finding suggests there is an environmental reservoir for norovirus, and that rain alters the turbidity of reservoirs of water-borne norovirus.

In the second place, it is notable that the relationship between rainfall and NAGO epidemic incidence involves a time lag between a period of higher rainfall and an eventual increase in NAGO incidence. The time lag was, on average, about three months. From a mechanistic viewpoint this observed time lag makes sense. It can be argued that if rain is required to facilitate the spread of environmental sources of norovirus then some time lag before there was a rise in NAGO incidence is reasonable. Implicit in this explanation is that there is a lengthy chain of events between a period of high rainfall and a NAGO epidemic so that it is not surprising that there is a variation in lag between one NAGO epidemic and the next.

The findings of the current study indicate rainfall can influence NAGO incidence. This suggests that changes in global weather patterns could ultimately influence patterns of norovirus epidemics.

## Figures and Tables

**Table 1:** The time lag between highest rainfall for each calendar year of the study (2002–2007) and NAGO epidemics.

**Year**	**3mth period of highest NAGOs**	**3mth period of highest rainfall**	**Midpoint-midpoint time delay (mths) for rainfall**
2002	Sep–Nov	May–Jul	4
2003	Oct–Dec	Jun–Aug	4
2004	Aug–Oct	Jun–Aug	2
2005	Oct–Dec	Jun–Aug	4
2006a	May–Jul	May–Jul	0
2006b	Oct–Dec	May–Jul	5
2007	Aug–Oct	May–Jul	3
